# Synthetic biology approaches for improving the specificity and efficacy of cancer immunotherapy

**DOI:** 10.1038/s41423-024-01153-x

**Published:** 2024-04-11

**Authors:** Bo Zhu, Hang Yin, Di Zhang, Meiling Zhang, Xiaojuan Chao, Luca Scimeca, Ming-Ru Wu

**Affiliations:** 1 Department of Liver Surgery, Center of Hepato-Pancreato-Biliary Surgery, Institute of Precision Medicine, The First Affiliated Hospital, Sun Yat-sen University, Guangzhou, 510080 China; 2 Department of Cancer Immunology and Virology, Dana-Farber Cancer Institute, Boston, MA 02215 USA; 3 Department of Immunology, Harvard Medical School, Boston, MA 02115 USA; 4 Drug Safety Research & Evaluation, Takeda Pharmaceuticals International Company, Cambridge, MA 02139 USA; 5 Medical Research Institute, Guangdong Provincial People’s Hospital, Southern Medical University, Guangzhou, 510080 China

**Keywords:** Synthetic biology, Cancer immunotherapy, Gene circuit, Gene therapy, Adoptive cell therapy., Tumour immunology, Immunotherapy, Applied immunology

## Abstract

Immunotherapy has shown robust efficacy in treating a broad spectrum of hematological and solid cancers. Despite the transformative impact of immunotherapy on cancer treatment, several outstanding challenges remain. These challenges include on-target off-tumor toxicity, systemic toxicity, and the complexity of achieving potent and sustainable therapeutic efficacy. Synthetic biology has emerged as a promising approach to overcome these obstacles, offering innovative tools for engineering living cells with customized functions. This review provides an overview of the current landscape and future prospects of cancer immunotherapy, particularly emphasizing the role of synthetic biology in augmenting its specificity, controllability, and efficacy. We delineate and discuss two principal synthetic biology strategies: those targeting tumor surface antigens with engineered immune cells and those detecting intratumoral disease signatures with engineered gene circuits. This review concludes with a forward-looking perspective on the enduring challenges in cancer immunotherapy and the potential breakthroughs that synthetic biology may contribute to the field.

## Introduction

A fundamental challenge in cancer therapies is to distinguish tumor cells from normal cells and target the detected tumor cells specifically. As tumor cells usually bear foreign (altered-self) antigens due to somatic mutations and immune cells can precisely target foreign antigens, the immune system can be harnessed to target cancer cells more specifically than traditional therapies that target less specific tumor signatures (e.g., chemotherapy targeting proliferation markers). The history of leveraging the immune system to treat cancer can be traced back to as early as 1891, when Dr. William Bradley Coley first attempted to actively trigger antitumor immune responses through intratumoral injections of inactivated bacteria, known as Coley’s toxins [1]. However, Coley’s toxins were not widely acknowledged at that time due to unclear mechanisms of action and the fear of the potential risks associated with pathogenic bacteria. After over a century of development, immunotherapy has revolutionized the field of cancer treatment, drawing considerable attention to its unique advantages over conventional approaches. Currently, a wide spectrum of immunotherapeutic strategies have been tested in clinical trials, and they have changed the landscape of cancer treatment. A milestone in cancer immunotherapy was the Food and Drug Administration (FDA) approval of the checkpoint inhibitor ipilimumab, a monoclonal antibody targeting cytotoxic T lymphocyte antigen 4 (CTLA-4) in 2011 [2], which paved the way for other immunotherapeutic strategies, such as the use of monoclonal antibodies targeting programmed cell death 1 (PD-1) or PD-1 ligand 1 (PD-L1) [3]. These checkpoint inhibitors work by blocking the interaction between PD-1 on T cells and PD-L1 on tumor cells, allowing the immune system to attack cancer cells more effectively. In addition to checkpoint inhibitors, the FDA has also approved chimeric antigen receptor (CAR)-T-cell therapies [4–6]. This type of therapy involves genetically modifying a patient’s T cells to express a CAR that targets a specific antigen on the surface of cancer cells. CAR-T-cell therapy has shown remarkable success in treating certain types of blood cancers. Furthermore, other classes of immunotherapies, including a range of cellular therapies, such as tumor-infiltrating lymphocytes (TILs) [7], lymphocyte-activating cytokines, immunostimulatory antibodies, oncolytic viruses, and cancer vaccines [8, 9], have also been under active development. The development of these novel immunotherapeutic strategies provides hope for cancer patients who may have exhausted traditional treatment options.

Despite substantial breakthroughs in the field of cancer immunotherapy, there are still several obstacles that hinder its safety and efficacy, impeding its further development. One such safety challenge is on-target off-tumor toxicity. As tumor-specific antigens are extremely rare, tumor-associated antigens (TAAs) are commonly used as CAR-T-cell targets. However, these targets are also expressed in healthy tissues and can cause CAR-T-cells to trigger severe on-target and off-tumor toxicity. For example, off-tumor targeting can result in the elimination of all normal B cells by CD19 CAR-T cells, which is generally tolerable and clinically manageable when used to treat B-cell malignancies [10–12]. However, off-tumor toxicity can cause lethality when treating solid tumors [13, 14]. Another safety challenge is potential severe systemic toxicity. For example, CAR-T cells can cause severe cytokine release syndrome, and checkpoint inhibitors can trigger autoimmune-like toxicities that affect multiple organs [15, 16]. These severe systemic toxicities limit the applications of immunotherapy.

In addition to safety concerns, achieving potent and long-lasting therapeutic efficacy presents another challenge. While hematological malignancies, such as acute lymphocytic leukemia and multiple myeloma, show robust initial responses to CAR-T-cell therapies, a majority of these patients still experience tumor relapse due to tumor heterogeneity and antigen loss [17]. Furthermore, due to the suppressive tumor microenvironment and inefficient immune cell infiltration to tumor sites, it is still challenging for immunotherapies to achieve robust efficacy in treating solid tumors [18]. Currently, no CAR-T-cell therapies have been approved for treating solid tumors, and there is a pressing need to develop such approaches [19, 20]. Many of the abovementioned key challenges have been excellently reviewed.

Synthetic biology-based methods enables the design and generation of living cells with customized functions. By adapting engineering principles such as modular design, standardized parts, and systemic simulation, new devices that perform sophisticated cellular functions can be developed. One area in which synthetic biology holds great promise is the creation of smarter cell therapies and gene therapies to treat cancers. In this review, we focus on synthetic biology approaches that improve the specificity, controllability, and efficacy of immunotherapy. We categorize the approaches into two major categories and discuss them separately: (1) approaches that target tumor surface antigens with engineered immune cells and (2) approaches that target intratumoral disease signatures with engineered gene circuits. Finally, we conclude the review by discussing our perspective on tackling outstanding challenges in cancer immunotherapy and the promise that synthetic biology brings to the field.

### Synthetic biology approaches targeting tumor surface antigens with engineered immune cells

CAR-T-cell therapy is an exemplary type of engineered immune cell-based therapy that targets tumor surface antigens. Upon direct recognition and binding of tumor antigens, such as cell-surface proteins, carbohydrates, and glycolipids [21, 22], CARs can mediate antigen-specific T-cell activation independent of peptide-MHC recognition [23]. The design of CAR-T cells has evolved rapidly due to advancements in protein engineering. All CARs contain an extracellular antigen recognition domain. The intracellular part of the first-generation CARs includes only an activation domain, CD3ζ, which results in a limited T-cell response. Subsequently, one or two costimulatory domains were added to create second-generation CARs and third-generation CARs, respectively. These modifications aimed to improve CAR-T-cell proliferation and persistence. In the fourth generation of CARs, known as armored CARs, additional modifications were made to enable the production of cytokines or to leverage the intracellular domains of cytokine receptors to further enhance antitumor efficacy [24–29]. In this paper, we will use CAR-T-cell therapy as an example to illustrate how the tools and design principles of synthetic biology can be applied to enhance the specificity and controllability of various treatment modalities.

#### Logic-gated CARs to alleviate off-tumor toxicity

Ideally, CAR-T cells should specifically recognize and target antigens that are exclusively present on tumor cells. However, it remains challenging to identify such an antigen target for most tumor types. As tumor-specific antigens are rare, most CAR-T-cell strategies target TAAs. However, TAAs are also expressed in some normal tissues, potentially causing severe on-target and off-tumor toxicity. For example, using anti-HER2 CAR-T cells to treat metastatic colon cancer has caused fatal toxicity, potentially due to the on-target and off-tumor interactions of CAR-T cells with lung tissues [30]. As it is more readily feasible to use combinatorial expression patterns of tumor antigens to distinguish tumor cells from normal cells and to minimize on-target and off-tumor toxicity, CAR-T cells have been designed to sense multiple antigen targets and integrate the detected signals with logic gates to enhance tumor-targeting specificity [31].

**Fig. 1:**
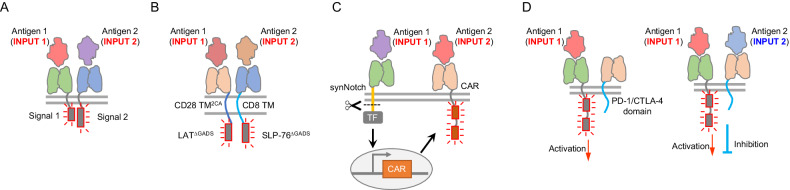
CAR designs that enhance tumor-targeting specificity. **A** An AND gate design based on splitting the T-cell activation motif (signal 1) and the costimulation motif (signal 2) into two tumor-targeting receptors. Only when the target cell expresses both antigens 1 and 2 to trigger both receptors, will the integrated signal fully activate T cells. **B** The design of the AND-gated LINK CAR. This design links the LAT protein to a scFv recognizing antigen 1 and the SLP-76 protein to another scFv recognizing antigen 2. Upon T cells encounter both antigens, LAT and SLP-76 interact and trigger T-cell activation. Several mutant variants have been designed to reduce on-target off-tumor toxicity, including cysteine residue mutations in the CD28 TM domain (2CA mutation) and GADS-binding site deletions in both LAT and SLP-76. **C** A synNotch receptor-based CAR design. The synNotch receptor is used to recognize antigen 1, which in turn activates the expression of CAR-targeting antigen 2. Therefore, only when the target cell expresses both antigens will the T-cell kill the target cell. **D** AND-NOT gate design. The activation CAR exhibits a standard CAR design and recognizes a tumor antigen (antigen 1). The inhibitory CAR (iCAR) recognizes a normal cell antigen (antigen 2) and delivers an inhibitory signal to T cells. Only when the target cell expresses antigen 1 but not antigen 2 will the AND-NOT gate trigger the T-cell to kill the target cell

One such strategy involves the use of AND gate circuits to improve the ability of CAR-T cells to specifically recognize and target tumor cells [32]. The AND gate circuits function by requiring the recognition of two or more antigens that are simultaneously presented on tumor cells but not on normal cells to activate CAR-T cells. Therefore, this strategy can prevent CAR-T cells from killing normal cells that express only one of the targeted antigens. Several types of AND gates have been developed for CAR-T cells. One common type utilizes two split CARs to recognize two antigens and achieve optimal tumor targeting. Contrary to the traditional second-generation CARs, this design explicitly separates the signaling motif (i.e., CD3ζ) that triggers signal 1 (antigen-recognition signal) and the signaling motif (i.e., CD28, 4-1BB) that triggers signal 2 (costimulation signal) of T-cell activation and links them with two different antigen recognition domains. After fine-tuning the affinity of the targeting scFv, this split CAR can specifically target tumor cells expressing both antigens and spare normal cells harboring only one of these target antigens (Fig. 1A). Several examples of CAR-T-cell designs that utilize such split-CAR strategies have been developed to improve the treatment of various cancer types. For example, one study utilized dual recognition of prostate stem cell antigen (PSCA) (with an anti-PSCA scFv-CD3ζ to provide signal 1) and prostate-specific membrane antigen (PSMA) (with an anti-PSMA scFv-CD28-4-1BB to provide signal 2) to target prostate cancer [32]. Another study utilized the dual recognition of mesothelin (with an anti-mesothelin scFv-CD3ζ to provide signal 1) and folic receptor alpha (FRα) (with an anti-FRα scFv-CD28 to provide signal 2) to target ovarian cancer [33]. These studies, along with other split CAR designs [34–37], demonstrate the potential of using AND gate circuits to alleviate the off-tumor toxicity associated with targeting TAAs and potentially extend the application of CAR-T cells to difficult-to-target tumor types.

Recently, another AND gate design based on coopting proximal T-cell signaling proteins to trigger CAR-T-cell activation has been developed [38]. This design utilizes the clustering of two proximal T-cell signaling proteins, linker for activation of T cells (LAT) and SH2 domain-containing leukocyte protein of 76 kDa (SLP-76), to construct an AND gate at the signal transduction level. By linking LAT to one scFv recognizing antigen 1 and SLP-76 to another scFv recognizing antigen 2, this dual-receptor design triggers strong T-cell activation when both receptors engage tumor antigens. In addition, to reduce single antigen leakage, the cysteine residues in the CD28 transmembrane domain (TM) domain were mutated (2CA mutation) to reduce heterodimerization and homodimerization. Furthermore, as the Grb2-related adapter protein downstream of Shc (GADS) inherently interacts with LAT and SLP-76 to form a scaffold for PLCγ, which activates downstream signaling pathways and can contribute to single antigen leakiness, the authors further removed the GADS-binding sites in both LAT and SLP-76 to enhance AND gate performance (Fig. 1B). This design can eliminate solid tumors specifically and effectively while substantially preventing off-tumor toxicity in vivo in mouse models [38].

Another AND gate was designed based on the synthetic Notch (synNotch) receptor system [39]. Briefly, the synNotch receptor is activated upon binding of the first antigen, which leads to the cleavage and release of a synthetic transcription factor (TF). This synthetic TF, in turn, can trigger the expression of a CAR that recognizes the second antigen. As a result, only tumor cells expressing both antigens trigger T-cell activation (Fig. 1C). In addition to expressing CAR constructs, this synthetic TF can also be used to trigger the production of additional payloads, such as cytokines and Toll-like receptor agonists, which can help to remodel the tumor microenvironment and improve antitumor efficacy [39–42]. In addition to being used for building AND gates, the synNotch receptor system has also been used for building a two-step positive-feedback circuit that allows T cells to discriminate targets on the basis of a sigmoidal antigen density threshold. This design is based on the use of a low-affinity anti-HER2 synNotch receptor to control the expression of a high-affinity anti-HER2 CAR for T cells. It allows T cells to exhibit sharp discrimination between off-target cells expressing normal amounts of HER2 and cancer cells expressing 100 times as much HER2 both in vitro and in vivo [43].

In addition to creating an AND gate that is activated by two TAAs, scientists have also devised AND-NOT gates that respond to the presence of a tumor antigen and the absence of a normal cell antigen. The NOT-gate part of the design is based on fusing an antigen-recognition receptor to the signal domain of immunoinhibitory receptors (e.g., PD-1 and CTLA-4) to create inhibitory chimeric antigen receptors (iCARs) (Fig. 1D). These iCARs can reversibly inhibit CAR-triggered T-cell activation. T cells expressing both the traditional CAR and the iCAR can perform tumor antigen AND-NOT self-antigen computation, sparing critical tissues expressing self-antigens to enhance targeting precision [44].

In summary, logic computations can enhance tumor-targeting precision and can be achieved with various types of designs [45]. For example, AND gates have been generated by signal integration [33], transcription cascades [39], receptor colocalization [46], and protein‒protein interactions [47]. The time required for completing logic computations will differ between various designs, and each design will also have a varied level of tunability and modularity. Thus, one open question remains: what is the optimal design for clinical translation?

#### Tunable switches to enhance the controllability of CAR-T cells

Once CAR-T cells are infused back into patients, the intensity of the immune response triggered by them and the extent of CAR-T-cell proliferation are usually beyond control. Rapid cytokine release and robust CAR-T-cell expansion can trigger severe toxicity, while the rapid clearance of a large number of tumor cells may induce tumor lysis syndrome [48]. However, substantial interindividual variability poses a particular challenge in predicting individual patient responses and associated toxicities. To mitigate these challenges, it is necessary to develop a controllable system that enables the fine-tuning of T-cell activity, which can substantially enhance the safety profile of CAR-T-cell therapy.

**Fig. 2:**
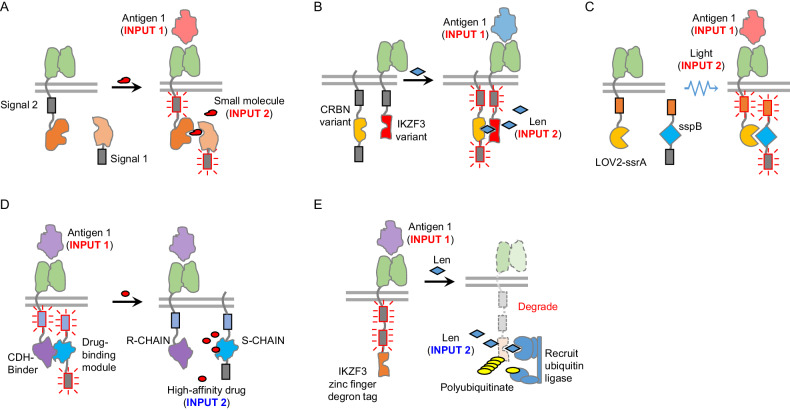
Tunable switches for controlling CAR-T-cell activity. **A** The design of an “ON-switch”. This type of design splits a conventional CAR into two individual components. Upon the administration of a small molecule drug, both components will assemble into a functional CAR and enable T cells to be activated by the target antigen. **B** An “ON-switch” CAR based on lenalidomide-induced receptor dimerization. Lenalidomide induces dimerization of an IKZF3 variant and a CRBN variant, leading to functional CAR formation that can be activated by the target antigen. **C** The design of the LiCAR. This design utilizes light-inducible dimerization of LOV2-ssrA and sspB to control the assembly of two separate CAR chains into a functional chain that can be activated by the target antigen. **D** The design of an “OFF” switch. This design consists of two separate CAR components and can naturally form a functional CAR. When a small molecule drug is administered, the functional CAR will disassemble and lose its function. **E** The design of an “OFF” switch based on small molecule-induced protein degradation. An IKZF3-based degron is tagged at the CAR. When lenalidomide is administered, it recruits the CRL4^CRBN^ E3 ubiquitin ligase to trigger the degradation of CAR

To address these issues, scientists have engineered an “ON switch” to regulate the activation and function of CARs in a tunable and reversible manner. This strategy involves the splitting of a conventional CAR construct into two separate components, which can be conditionally reassembled into a functional heterodimer by a small molecule. In one such design, one component includes an antigen-binding domain, an intracellular costimulation domain, and an interaction domain. The other component contains an interaction domain and immunoreceptor tyrosine-based activation motifs (ITAMs) from the T-cell receptor CD3ζ subunit. The small molecule induces the reconstitution of a functional CAR for T-cell activation (Fig. 2A) [49]. With this design, CAR-T-cell activity could be controlled by the administration of various doses of small molecules. Several chemically inducible dimerization pairs are commonly used, including rapamycin-induced dimerization of FK506-binding protein (FKBP) and FKBP-rapamycin-binding protein (FRB) [50], abscisic acid (ABA)-induced dimerization of ABA insensitive 1 amino acids 126 to 423 (ABI_cs_) and PYL1 amino acids 33 to 209 (PYL_cs_) [51], and gibberellin-induced dimerization of gibberellin insensitive dwarf1 (GID1) protein and gibberellin insensitive (GAI) protein [52]. In addition, new pairs of dimerization domains have been developed. For example, an IKZF3 variant and its interaction partner, a mutated CRBN that cannot bind to damage specific DNA binding protein 1 (DDB1) and lacks E3 ubiquitin ligase activity, were engineered with enhanced affinity to interact with the dimerization agent lenalidomide. A recently developed split CAR, which contains two components, utilized this interaction pair [53]. Specifically, one component is a fusion protein of an antigen receptor, a signaling 2 (i.e., CD28) domain, and the IKZF3 variant, and the other component is a fusion protein of a mutated CRBN and a signal 1 (i.e., CD3ζ) domain (Fig. 2B). Notably, this “ON-switch” design was able to accomplish drug-dependent antitumor activity in vivo [53].

Light-inducible CARs (LiCARs) have also been devised to achieve spatiotemporal control of CAR-T-cell activity [54]. This design utilizes the light-dependent interaction of the fusion protein LOV2-ssrA and sspB. Similarly, the CAR was split into two separate chains. One chain contains an antigen recognition domain, a costimulatory domain, and LOV2-ssrA. The other chain contains sspB and a CD3ζ activation domain. T-cell activation requires antigen engagement and light induction to mediate the dimerization of the two chains (Fig. 2C). By using an upconversion nanoplate as a miniature light transducer to emit deep tissue-penetrable near-infrared light, one can activate these LiCAR T cells in vivo [54].

High-frequency focused ultrasound can also be used to control CAR expression. The mechanosensor Piezo1, which can respond to ultrasound stimulation to trigger calcium influx and activate calcium-sensitive cascades, was used to drive CAR expression in engineered T cells, providing remote and noninvasive control of cancer immunotherapy [55]. In addition, in a recent study, the expression of the CAR was shown to be controlled by the promoter of the heat shock protein. In mice with subcutaneous tumors, locally injected CAR-T cells can suppress tumor growth and mitigate on-target off-tumor activity if activated via focused ultrasound guided by magnetic resonance imaging [56]. In another study, CAR-T cells were loaded with magnetic beads coated with anti-CD3/CD28 antibodies for magnetic-acoustic actuation. The sequential magnetic and acoustic actuation allowed CAR-T cells to migrate and penetrate deep into tumor tissues. The anti-CD3/CD28 immunomagnetic beads further activated the infiltrating T cells and enhanced their antitumor efficacy.

In addition to “ON-switch” strategies, “OFF-switch” strategies have also been designed to control CAR-T-cell activity. These designs create a controllable system that can turn off CAR-T-cell activity when necessary. One such design incorporates small-molecule controllable elements in the split CAR. This split CAR consists of two chains, one containing an antigen-recognition domain fused with a CD3ζ signaling domain and the other containing a CD28 signaling domain (Fig. 2D). To facilitate the small-molecule regulation of CAR-T-cell activity, a heterodimeric domain pair was integrated into the cytoplasmic domain of each chain of the split CAR. As a result, the split CAR can automatically dimerize and form a functional CAR. However, by using a small molecule, this interaction can be disrupted, thereby inhibiting CAR function. This design exhibits antitumor activity comparable to that of second-generation CAR-T cells in the absence of disruptive agents, and the administration of disruptive agents has been shown to inhibit the activity of CAR-T cells both in vitro and in vivo [57].

Besides interrupting the physical interactions of the split receptors, CAR degradation represents another strategy to halt CAR-T-cell activities. To accomplish this goal, scientists have utilized a drug-inducible degron, which serves as an “OFF switch” for CAR-T cells. Specifically, a design utilizing a lenalidomide-inducible degron has been developed in which the degron is attached to the C-terminus of the CAR. When thalidomide or its analog lenalidomide is administered, it acts as a molecular glue to bridge the CRL4^CRBN^ E3 ubiquitin ligase and the degron, which triggers the degradation of CAR both in vitro and in vivo (Fig. 2E) [53].

In addition, OFF switches that trigger apoptosis have also been developed. In one study, a fusion gene consisting of the apoptotic gene Caspase 9 and the human FK506-binding protein FKBP12 was introduced into T cells. After dimerization with AP1903, the Caspase 9 protein can trigger apoptosis [58]. This design allowed researchers to eliminate more than 90% of the modified T cells within 30 min after drug administration [58]. Currently, CAR-T cells equipped with this irreversible OFF switch have been tested in several clinical trials (e.g., NCT03016377, NCT03696784, NCT02107963, and NCT03721068).

In summary, controlling the intensity of the immune response triggered by CAR-T-cell therapy is another strategy for enhancing its safety profile. Tunable switches have been accomplished by leveraging small molecule-induced split CAR dimerization [49], CAR degradation [53], CAR expression [59] and cellular apoptosis [58]. Some of these switches can also be used to regulate CAR expression to enable T cells to rest, which in turn prevents T-cell exhaustion [60]. Each design has a different tunable dynamic range and kinetics. Other than the inducible Caspase 9 system, most of the designs are at preclinical development stages and have not yet been tested in clinical trials. One open question remains: what is the optimal design for clinical translation?

#### CAR designs that can mitigate tumor antigen loss

The effectiveness of CAR-T-cell therapies is significantly hindered by antigen expression heterogeneity and the downregulation of antigen expression in tumor cells under therapeutic intervention. Such antigen loss can severely compromise therapeutic efficacy. In addition, due to the laborious and expensive manufacturing process of CAR-T cells, it is infeasible to recreate a new CAR for every instance of tumor relapse. Consequently, developing strategies that can overcome potential antigen loss during therapy is critical for the clinical success of CAR-T-cell therapies.

**Fig. 3:**
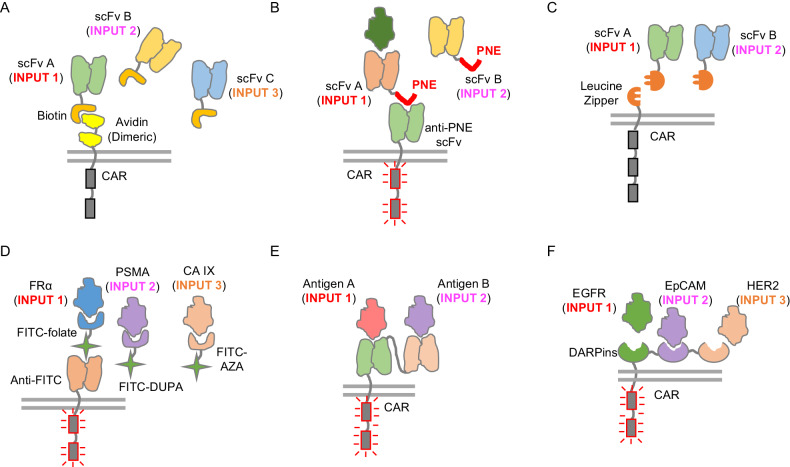
CAR designs that can mitigate tumor antigen loss. **A** The design of the biotin-binding immune receptor (BBIR) CAR. BBIR can bind to the administered scFv-biotin molecule and therefore enable T cells to target a specific tumor antigen. By administering different scFv-biotin molecules, BBIR CARs can target a variety of antigens. **B** The design of an anti-PNE CAR that works with the administration of a PNE-fused tumor-targeting domain. By utilizing different PNE-fused tumor-targeting molecules, the anti-PNE CAR can target various tumor antigens. **C** The design of the SUPRA CAR. This system requires the expression of a universal receptor (zipCAR) expressed on T cells and the administration of a tumor-targeting adapter molecule (zipFv). The two leucine zippers will allow a functional CAR to assemble, which can then be activated by tumor antigens. By administering different zipFv molecules, the SUPRA CAR can target different antigens. **D** The design of an anti-FITC CAR for targeting multiple tumor antigens. This system requires the expression of an anti-FITC CAR on T cells and the administration of FITC-conjugated tumor-targeting moieties such as FITC-folate, FITC-DUPA and FITC-AZA adapter, which enable T cells to target antigens such as FRα, PSMA and CA IX on tumor cells, respectively. **E** The design of a bispecific CAR. This design links two tumor-targeting scFvs in tandem for targeting two tumor antigens. **F** The design of a multispecific CAR. This design links three DARP proteins in tandem to target three different tumor antigens

A potential solution to this issue involves targeting multiple tumor antigens concurrently to enhance therapeutic efficacy. One such design replaces the extracellular antigen-binding domain of a conventional CAR with a biotin-binding domain (i.e., avidin). Upon the administration of a scFv-biotin fusion molecule, this molecule can bind to engineered T cells and provide them with tumor-targeting ability (Fig. 3A) [61]. This system enables the targeting of various tumor antigens by administering different scFv-biotin fusion molecules. Another strategy involves replacing the conventional CAR’s extracellular antigen-binding domain with a scFv that recognizes PNE, a 14-amino acid peptide sequence. This system can be directed to target multiple antigens by administering a range of tumor-antigen targeting domains fused with the PNE. This strategy can potentially overcome the heterogeneity of tumor antigen expression and prevent antigen loss-mediated escape (Fig. 3B) [62].

Another design that operates on a comparable principle is referred to as the “SUPRA CAR” system. This approach necessitates the genetic modification of T cells to express a universal receptor, known as zipCAR, followed by the administration of tumor-targeting scFv molecules, called zipFv. The zipCAR includes a leucine zipper (zip) positioned at the extracellular domain of the CAR, while the zipFv molecule contains a tumor-targeting scFv and a matching leucine zipper that can form a heterodimer with the zipCAR. The SUPRA CAR system has been used to implement an OR gate to target both Axl and Her2 to demonstrate the potential of this system to overcome antigen escape (Fig. 3C) [63].

Similarly, in another study, an anti-FITC CAR was expressed on T cells, and a cocktail of orthogonal FITC-linked bispecific adapters (i.e., FITC-folate, FITC-DUPA, and FITC-AZA) was administered to target various tumor antigens, such as FRα, PSMA and CA IX. The antigen-targeted end of this adapter binds to tumor antigens that are highly expressed in many human cancer cells and minimally expressed in normal tissues [64–66]. The FITC end binds to the anti-FITC CAR to connect the T cells with the tumor cells (Fig. 3D) [67]. This system can direct T cells to target multiple tumor antigens, potentially overcoming tumor antigen escape in CAR-T-cell therapy. Furthermore, by adjusting the dosage of the adapter, one can regulate CAR-T-cell activity and potentially prevent cytokine release syndrome-like toxicity [68].

In addition, bispecific CAR-T cells have been developed that use two tandem scFvs to target two antigens or two epitopes on the same antigen simultaneously (Fig. 3E) [69–75]. Some of these bispecific CARs have been tested in clinical trials. For example, a tandem CAR with two antigen binding domains for targeting two BCMA epitopes has gone through various clinical trials and has been FDA-approved [73, 74, 76, 77]. This CAR design can elicit robust and durable responses with a manageable safety profile in relapsed/refractory multiple myeloma [73, 74]. Another tandem CAR targeting both CD20 and CD19 has demonstrated a manageable safety profile and high efficacy in a phase I clinical trial against adult patients with B-cell non-Hodgkin lymphoma or chronic lymphocytic leukemia, providing support for mitigating tumor antigen loss and relapse [75]. Other bispecific CARs under clinical trials include anti-CD19/CD22 (NCT03241940, NCT04303520, and NCT05523661), anti-CD19/BCMA (NCT03879382 and NCT03706547), and anti-CS1/BCMA (NCT04662099 and NCT05950113). These studies highlight the translational potential of tandem CARs in the clinic. However, encoding multiple scFvs in tandem can sometimes be challenging due to the increased vector size and potential for antibody aggregation [78]. As a result, an alternative antibody mimetic framework, designed ankyrin repeat proteins (DARPins), has been explored to construct tandem CARs [79]. DARPins are small engineered proteins that consist of ankyrin repeat modules, with each module comprising 33 amino acids [80]. These proteins exhibit specificities and affinities comparable to those of antibodies, enabling them to recognize target antigens with high precision [81]. It has been demonstrated that trispecific CARs constructed with tandem DARPins can target heterogeneous tumors and display synergistic activity against tumor cells that express multiple antigens in vivo (Fig. 3F) [79].

In summary, most of the abovementioned systems necessitate the creation of an adapter protein for T cells in conjunction with the provision of a molecule that targets tumors. While such augmented complexity can enhance targeting versatility, it can also potentially hamper clinical translation. Therefore, one open question remains: what is the optimal strategy for striking a balance between versatility and complexity?

### Synthetic biology approaches targeting intracellular tumor signatures with engineered gene circuits

As most oncogenic proteins and tumor signatures are located inside tumor cells, synthetic biology approaches that target intracellular tumor signatures have the potential to synergize with cell therapy strategies that target cell surface proteins. These approaches can be broadly organized into the following categories.

#### Strategies for detecting aberrant oncogenic signaling pathways

**Fig. 4:**
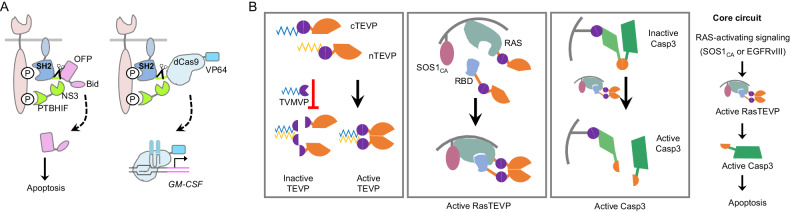
Synthetic gene circuits for detecting intracellular signaling. **A** Design of the RASER system. This system contains two components. One component is a fusion protein consisting of a membrane-tethered SH2 domain, an NS3 cleavage site, and a therapeutic payload [e.g., OFP-Bid (left panel) or dCas9-VP64 (right panel)]. The other component is a fusion protein consisting of a PTB domain, a HIF1α degron, and an NS3 protease domain. In ErbB-hyperactive tumor cells, the two components colocalize, and the NS3 protease in one component cleaves its target site on the other component, releasing the therapeutic payload to induce apoptosis (left panel) or to activate the transcription of endogenous genes such as GM-CSF (right panel). **B** The design of the CHOMP system. This system utilizes interacting synthetic proteases for computations. For example, TEVP can be split into two halves (cTEVP and nTEVP) and can function as a protease by interacting with a pair of leucine zippers. A synthetic TVMVP can be designed to abrogate the reconstitution of the cTEVP and the nTEVP, inhibiting the function of TEVP. These interacting proteases can be used to accomplish various logic computations. CHOMP requires two components to detect the upstream signals that activate the RAS. One component consists of the RAS protein fused with the nTEVP, and the other component consists of a RAS-binding domain (RBD), which binds to the active form of RAS fused with the cTEVP. The two components are reconstituted together in cells with high RAS-activating signals and form a functional TEVP. This TEVP further cleaves its substrate sequence and releases active Caspase 3 (Cas p3) from a membrane-anchored complex to trigger apoptosis

A system called Rewiring of Aberrant Signaling to Effector Release (RASER) has been designed to sense an intracellular ErbB oncogenic signal and rewire this signal to trigger programmable therapeutic outputs [82]. In certain types of cancer, ErbB is constitutively activated and phosphorylated at cytoplasmic tyrosine residues. As phosphorylated ErbB can be bound by the phosphotyrosine-binding (PTB) and SH2 domains [83], the RASER system leverages these interactions with two engineered protein components. One component is a fusion protein consisting of a cell membrane tethered-SH2 domain, an NS3 protease cleavage site, and a therapeutic cargo. Examples of cargos include the Bid protein, which can induce apoptosis, and dCas9-VP64, which can activate gene expression. The other component is a fusion protein consisting of a PTB domain, a HIF1α degron, and an HCV NS3 protease domain. In ErbB-hyperactive cells, both components are simultaneously recruited and colocalized at activated ErbB receptors. This colocalization enables the NS3 protease to cleave its substrate sequence and release therapeutic cargos (Fig. 4A). RASER can preferentially target ErbB-hyperactive cancer cells for apoptosis and CRISPR/Cas9-mediated specific gene expression while sparing ErbB-normal cells in vitro [82].

Another system called Circuits of Hacked Orthogonal Modular Proteases (CHOMP) utilizes synthetic proteases for computation and can achieve a variety of circuit-level functions in mammalian cells [84]. The design utilizes several viral proteases that exhibit strong specificity for short cognate cleavage sites. For example, tobacco etch virus protease (TEVP) can be cleaved and then reconstituted through the dimerization of leucine zippers. A leucine zipper-tagged tobacco vein mottling virus protease (TVMVP) can dock with the target TEVP and cleave it to remove the leucine zippers, leading to the loss of TEVP function (Fig. 4B). The CHOMP system was adapted to induce cell death in response to RAS activation. RAS activation allows CHOMP to form an active RasTEVP with a functional TEVP, which can then cleave its target sequence to release activated Caspase 3 from the membrane-attached complex to trigger apoptosis. This CHOMP circuit has the potential to detect and kill cancer cells in response to upstream activators of the RAS and may be further engineered to target other aberrant signaling pathways in tumor cells [84].

#### Strategies for sensing the TF activity profile

Tumor cells usually exhibit aberrant signal transduction pathways that converge into TF activity. Therefore, highly tumor-specific TFs can serve as therapeutic targets [85]. In addition, many cancer types share a core set of dysregulated master TFs [86]. However, it remains challenging to directly target TFs with small molecules because TFs are structurally dynamic [86]. Rather than targeting TFs directly with small molecules, synthetic biologists have developed strategies that sense such aberrant TF activities and convert the detected activities to therapeutic responses. Here, we focus on synthetic biology approaches that utilize promoters to detect aberrant TF activities for targeting cancers.

Utilizing promoters that are responsive to tumor-specific TFs is a potential solution for detecting overexpressed TFs in tumor cells and inducing therapeutic outcomes. Currently, a few cancer-specific gene therapies that utilize native tumor-specific promoters have been tested in clinical trials. A strategy that utilizes plasmids consisting of a tumor-specific promoter, H19, to express diphtheria toxin A has been tested in two phase II trials (NCT03719300 and NCT01878188). This strategy has been used as a single therapy or in combination with the BCG vaccine for treating bladder cancers.

Another study utilized an adenoviral vector consisting of an angiogenic endothelial cell-specific promoter (modified preendothelin 1 promoter) to drive the expression of the chimeric death receptor TNFR1-FAS. This strategy (VB-111) aims to induce tumor vascular disruption and an intratumor immune response [87]. When VB-111 is used in combination with paclitaxel, it is well tolerated and has demonstrated favorable response rates and improved survival outcomes for platinum-resistant ovarian cancer patients in various clinical trials (NCT01711970, NCT01260506, NCT01229865, and NCT04166383). However, in a recently reported randomized controlled phase III trial (NCT03398655), the addition of VB-111 to paclitaxel did not improve progression-free survival or overall survival in platinum-resistant ovarian cancer patients [88].

**Fig. 5:**
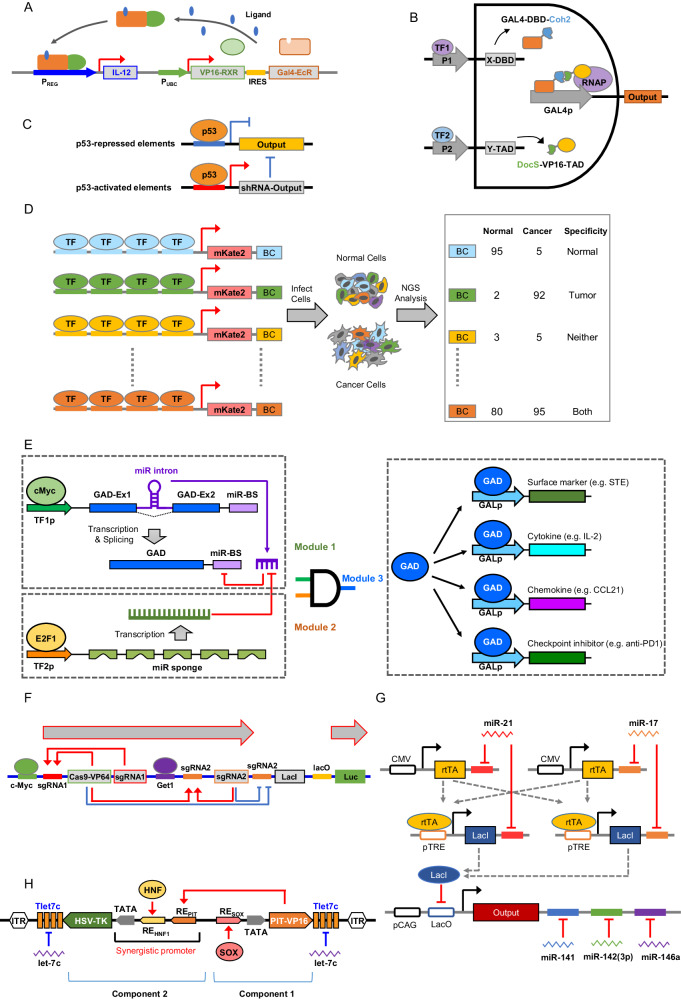
Synthetic gene circuits for detecting transcription factor activities or microRNA expression profiles. **A** The design of the inducible RheoSwitch® Therapeutic System (RTS). The Gal4-EcR and VP16-RXR fusion proteins are expressed constitutively under the Ubiquitin C promoter. The activating ligand will enable both fusion proteins to form functional heterodimers and therefore activate the transcription of IL-12. **B** The design of the Dual Promoter Integrator. To accomplish AND gate computation, two native tumor-specific promoters are used as tumor sensors to drive the expression of two protein components. One component is a fusion protein consisting of the GAL4 DNA binding domain fused with Coh2 (GAL4-DBD-Coh2). The other component is a fusion protein consisting of the VP16 transcription activation domain fused with DocS (DocS-VP16 TAD). When both components are expressed, the interaction of Coh2 and DocS will form a functional GAL4-VP16 complex to activate the output expression. **C** The design of a circuit that targets cells with low p53 activity. This design contains two modules. One module utilizes p53-repressed elements to suppress the expression of the output (i.e., HSV-TK). The other module utilizes p53-activated elements to trigger the production of shRNA that depletes the output. Therefore, this circuit will express high levels of HSV-TK when p53 activity is low. **D** The design of synthetic promoters. A synthetic promoter is usually built by fusing one type of TFBS in tandem upstream of a minimal promoter. Various synthetic promoter libraries have been built according to this design principle. High-throughput promoter activity analysis can be achieved by leveraging FACS sorting and next-generation sequencing analysis. **E** The design of an RNA-based immunomodulatory gene circuit for cancer immunotherapy. This circuit senses the activities of c-Myc and E2F1, two cancer-associated TFs, and triggers therapeutic output production only when both activities are high. Specifically, module 1 senses the activity of c-Myc and utilizes this activity to trigger the transcription of GAD, a synthetic TF, along with a microRNA (miRNA) that inhibits GAD transcript accumulation. Module 2 senses the activity of E2F1 and utilizes this activity to trigger the expression of a miRNA “sponge” that titrates the inhibitory miRNA. Hence, only when both c-Myc and E2F1 are highly active (AND gate) will  GAD accumulate and drive the production of the therapeutic outputs encoded in module 3. This circuit enables tumor-localized combinatorial immunotherapy. GAD: a fusion protein consisting of the GAL4 DNA binding domain and the VP16 transcription activation domain. STE: surface T-cell engager (a potent synthetic universal tumor antigen). **F** The design of a mini gene circuit based on CRISPReader. The c-Myc- and Get1-responsive elements are used to regulate the expression of the Cas9-VP64 protein and two sgRNAs (sgRNA1 and sgRNA2). Cas9-VP64 complexed with a 14 nt long sgRNA exhibited transcriptional activation only. When the activities of both c-Myc and Get1 are high, Cas9-VP64 and the two sgRNAs are produced. The produced Cas9-VP64 and the two sgRNAs can further form transcription-activating complexes to activate transcription. As there are sgRNA binding sites immediately after the c-Myc and Get1 responsive elements, the transcription-activating complexes further enhance the production of Cas9-VP64 and the two sgRNAs, forming a positive feedback loop. In addition, the expressed Cas9-VP64 and sgRNA2 can form a gene knockout complex to knock out the LacI gene. Knocking out the LacI gene will then enhance the final output production. As a result, only when the activities of both c-Myc and Get1 are high will the circuit trigger high-output production. **G** The design of the HeLa cell classifier. This classifier circuit contains sensors for both HeLa-high (miR-21 and miR-17) and HeLa-low (miR-141, miR-142(3p), and miR-146a) miRNAs. In HeLa cells where HeLa-high miRNAs are abundant, the production of rtTA and LacI will be low, and therefore, the circuit will trigger high therapeutic output. Furthermore, in HeLa cells where HeLa-low miRNAs are scarce, the therapeutic yield will remain high. **H** The design of a “SOX9/10 AND HNF1A/B AND (NOT let-7c)” gate for targeting HCC. This design consists of two transcription components. The first component can sense SOX9 or SOX10 and trigger the expression of a transactivator (PIT-VP16). The second component needs to sense both PIT-VP16 and HNF1A/B to trigger the expression of HSV-TK. Therefore, only when both SOX9/10 and HNF1A/B are expressed will HSK-TK be produced. The binding sites for highly expressed miRNAs in normal tissue (e.g., let-7c) were also incorporated into the 3’-UTRs of both the PIT-VP16 and HSV-TK genes to decrease off-target effects on normal cells. Abbreviations: ITR, inverted terminal repeat; RE_SOX_, binding site for SOX9/10; RE_HNF1_, binding site for HNF1A/B; RE_PIT_, binding site for PIT-VP16 or PIT-RelA; Tlet7c, four repeats of a fully complementary let-7c target

In addition, cancer-localized gene therapy with inducible switches has also been tested in clinical trials. A preclinical study utilized an adenovirus expressing an inducible IL-12 cassette (Ad-RTS-IL-12) to treat glioma. With this strategy, IL-12 production by tumor cells can be induced by the oral administration of veledimex [86] (Fig. 5A). The production of IL-12 can trigger immune cell infiltration into glioma tumor masses, supporting the immunological antitumor effect of IL-12 [89, 90]. This strategy was further tested in a clinical trial for the treatment of high-grade glioma (NCT02026271). In this trial, Ad-RTS-IL-12 was injected into the walls of the resected tumor cavity and showed acceptable tolerability to regulated IL-12, with encouraging preliminary effects [91]. More trials are ongoing to further evaluate this strategy in multiple tumor types (NCT01397708 and NCT02423902). These studies demonstrate that leveraging tumor-specific transcription factors as disease signatures is clinically translatable.

However, finding a single tumor-specific promoter that can effectively differentiate tumor cells from normal cells remains a challenge. To overcome this issue, a dual promoter integrator has been developed to merge promoter activity with a logical AND gate, which is executed by a pair of fusion proteins [92]. One of the fusion proteins is composed of a GAL4 DNA binding domain that is fused with Coh2 (GAL4-DBD-Coh2). The other fusion protein consists of a VP16 transcription activation domain that is fused with DocS (DocS-VP16-TAD). The expression of both fusion proteins is controlled by a set of two tumor-specific promoters. When both promoters are active, a functional GAL4-VP16 protein will be assembled through the interaction of Coh2 and DocS, leading to the expression of downstream outputs specifically in tumor cells (Fig. 5B). The dual promoter integrator can enhance the targeting precision compared to the use of a single promoter [92].

Furthermore, the loss of tumor suppressor TF activity can also be exploited to differentiate between tumor cells and normal cells. One important tumor suppressor is the p53 protein, which controls cell proliferation by promoting apoptosis and DNA repair. Mutations that affect p53 function can cause abnormal cell growth and tumor progression [93], making p53 a potential target for synthetic biology approaches. A gene circuit was constructed to sense the loss of p53 activity and specifically target cells lacking p53 activity (Fig. 5C). This circuit utilizes two promoters to regulate the production of a killer protein, herpes simplex virus thymidine kinase (HSV-TK). One promoter contains p53-repressible elements that trigger the production of HSV-TK when the cell does not have high p53 activity. The other promoter contains p53-inducible elements that trigger the production of shRNA targeting HSV-TK when the cell has high p53 activity. As a result, this circuit expresses a high level of HSV-TK when p53 activity is low. Upon the administration of ganciclovir, cells containing HSV-TK are eliminated; therefore, this circuit can selectively target tumor cells with p53 loss-of-function mutations both in vitro and in vivo [94]. However, because p53 activity in different normal cells varies, further studies are required to determine whether physiological p53 levels are high enough to suppress the production of HSV-TK.

Native promoters typically contain multiple types of TF-binding sites (TFBSs) and therefore can be activated by a variety of TFs. Therefore, native promoters are usually not completely “OFF” in normal cells and can trigger leaky expression of therapeutic outputs. To potentially overcome this hurdle, synthetic promoters containing only one type of TFBS have been developed with the aim of achieving a higher tumor-to-normal-cell activity ratio [95]. Indeed, such designs can usually achieve a higher tumor-to-normal-cell activity ratio [95]. Typically, synthetic promoters are composed of a series of identical TFBS sequences in tandem upstream of a minimal promoter. This design achieves even and dense distribution of the TFBSs within the promoter region. In addition, this design has inspired the development of synthetic promoter libraries that cover comprehensive TFBS motifs [95–97]. These libraries can be utilized to identify tissue-specific and cell state-specific promoters and have important utility for tissue-specific or disease-specific gene therapies (Fig. 5D) [95].

Furthermore, these de novo-created tumor-specific synthetic promoters have also been utilized to construct gene circuits aimed at modulating the immune response for treating cancer. For example, one study utilized these promoters as sensors to construct an RNA-computation-based AND gate circuit. If both promoters are active, the circuit will trigger a tumor-localized combination immunotherapy [98]. Specifically, this circuit contains three modules. Module 1 utilizes a tumor-specific synthetic promoter consisting of c-Myc binding sites to simultaneously regulate the production and degradation of a synthetic transcription factor, Gal4-VP16 (GAD). Module 2 utilizes another tumor-specific synthetic promoter consisting of E2F1 binding sites to regulate the production of a microRNA sponge that can inhibit the degradation of GAD. Therefore, only when both promoters are active will GAD be produced at a high level, which will then trigger the production of therapeutic outputs that are encoded in module 3 (Fig. 5E) [98]. In theory, this circuit can trigger combination immunotherapies of any genetically encodable protein with therapeutic effects. By triggering the combination of a universal tumor antigen, chemokine, cytokine, and checkpoint inhibitor, this circuit significantly reduces the tumor burden and prolongs survival in ovarian cancer mouse models. Importantly, even when only a small fraction of tumor cells were delivered via the circuit, a significant reduction in tumor burden and enhanced mouse survival were observed. This observation indicates that a cancer-targeting gene circuit that effectively triggers an immune response may not require high-efficiency circuit delivery to mediate robust efficacy.

Another study utilized the TF-binding elements of c-Myc and Get1 in the native promoters of the human uroplakin II gene and the telomerase reverse transcriptase gene, respectively, to construct two tumor-specific promoters. The former promoter drives the expression of Cas9-VP64 and a sgRNA (i.e., sgRNA1), while the latter drives the expression of another sgRNA (i.e., sgRNA2) for building a gene circuit (Fig. 5F). Cas9-VP64 can switch between DNA cleavage and transcriptional activation according to the length of the sgRNA, and Cas9-VP64 complexed with a 14 nt long sgRNA was shown to only activate transcription [99, 100]. Therefore, this circuit will only trigger high-output production when both promoters are active. As a result, high levels of both c-Myc and Get1 in bladder cancer cells drive the expression of Cas9-VP64 and both sgRNAs. The expressed sgRNAs can further bind to Cas9-VP64 to enhance their own production through a positive feedback mechanism. In addition, sgRNA2 can knock out the LacI repressor, which inhibits the production of the final output (Fig. 5F) [101]. This circuit can also be used to knock out endogenous genes. By using this system to overexpress p21 and E-cadherin, a decreased tumor burden was observed in vivo in a mouse model of metastatic bladder cancer.

#### Strategies for sensing endogenous microRNA (miRNA) signatures

In addition to dysregulated TF activity, aberrant miRNA expression is also linked to tumorigenesis and can be used as a biomarker to distinguish various tumor types [102]. A study built a gene circuit called the “HeLa classifier” to leverage the expression pattern of a set of miRNAs to classify various tumor types [103]. The HeLa classifier only triggers the output production when the cellular miRNA expression pattern matches a predetermined profile. Specifically, only when the expression levels of two predetermined miRNAs (miR-21 and miR-17-30a) are high and three miRNAs are low (miR-141, miR-142(3p), and miR-146a) will the HeLa classifier trigger output production (Fig. 5G) [103]. The HeLa classifier has been shown to specifically trigger apoptosis in HeLa cells by expressing Bax, an apoptosis-inducing protein, as a therapeutic agent. In addition, modeling-based circuit-topology selection and input optimization have also been created to automate the development of classifiers to target several cell types with optimal responses [104].

#### Strategies for simultaneously sensing TF activity and endogenous miRNA signatures

Another study built a compact cell classifier that utilizes multiple inputs for specifically targeting hepatocellular carcinoma (HCC) [105]. In this classifier, two HCC-specific synthetic promoters are used to construct a transcription-based AND gate. Specifically, this AND gate consists of two components. The first component utilizes SOX9/10 response elements to trigger the production of a pristinomycin-inducible transactivator (PIT). The second component utilizes HNF1A/B response elements and PIT response elements to drive output production. Therefore, only when both circuit components are actively transcribed will the circuit trigger high-output production. In addition, to further improve the targeting precision, miRNA binding sites for highly expressed miRNAs in hepatocytes and some normal tissues (e.g., miR-122, miR-424, let-7c) are introduced to the circuit to decrease off-target effects in normal cells. Finally, an optimized classifier employing “HNF1A/B AND SOX9/10 AND (NOT let-7c)” logic is used to distinguish HCC cells from normal cells and to express HSV-TK (Fig. 5H). Upon administration of ganciclovir, cells containing HSV-TK are eliminated.

In summary, various genetic circuits have been developed to detect aberrant TF activity, miRNA abundance, and oncogenic protein activity. These tumor signatures can also be used jointly as inputs for circuits. Depending on the signatures of interest, the design of tumor sensors and logic computation devices usually varies. As each type of sensor has intrinsic differences in sensitivity, each type of logic computation device has intrinsic differences in tunability and modularity, and certain signatures may be more clinically identifiable than others. One open question remains: is there an optimal type of signature or signature combination that is most clinically actionable? Moreover, as circuits have been developed to trigger immune responses, apoptotic pathways, and prodrug production, one open question remains: is there an optimal type of output or output combination that is most suitable for eliminating cancer cells and preventing tumor relapse?

## Conclusion and future perspectives

Synthetic biology approaches have brought immense potential and momentum to cancer therapy. Currently, most of the technologies described in this review have shown proof-of-concept results in experimental systems but have not been translated into the clinic. Translating these approaches to the clinic to obtain real-world efficacy data will provide important insights to move the field forward. To bridge the gap between proof-of-concept and clinical translation, gaining clarity in the following key aspects would be helpful for designing therapies.

1. What are the specific therapeutic functions or controls aimed to achieve?
  Synthetic biologists have a variety of tools to manipulate and control cellular behaviors and therefore often strive to implement multiple functions in their designs. Indeed, to perform more functions, a more complex circuit is usually needed. However, it is generally more difficult to construct a highly complex circuit for the final drug because multiple lentiviruses or gene-editing reagents are required for this process. In addition, therapeutic products are usually less homogeneous and are not preferred from a regulatory perspective. It will be critical to justify the added benefits outweighing the increased complexity. Reaching a fine balance will be necessary for clinical translation.
2. How targeted should synthetic biological approaches be?
  Since there are few, if any, perfect tumor signatures, synthetic biology designs usually integrate multiple tumor signatures with logic gates to distinguish tumor cells from normal cells. Utilizing multiple tumor signatures can indeed achieve increased targeting specificity. However, it may also allow more space for tumor cells to escape from being targeted. For example, when using an AND gate to target tumor cells, tumor cells are no longer targeted if they lose any of the targeted signatures. Thus, it will be critical to find the ideal balance. In addition, tumor cells are usually heterogeneous in patients. However, circuits are usually developed and optimized with relatively homogeneous in vitro tumor cell culture models. Hence, combining various model systems is needed to create clinically relevant gene circuits.
3. Which approach should be leveraged to achieve the above functionality?
  In this review, we primarily discuss cell therapy and gene circuit therapy. It remains unclear whether one approach is superior to the other or whether a preferred strategy should be adopted for specific tumor types. Additionally, it is worth investigating the potential benefits of leveraging both strategies and whether the complexity of the final therapeutic product can be justified. To obtain more clarity on these issues, it is imperative to collect real-world feasibility and efficacy data.

Finally, we also outline the following domain-specific opportunities.

### Opportunities in synthetic biology-inspired CAR-T-cell therapy

Currently, a plethora of gene circuits have been developed to improve the targeting precision and safety of CAR-T cells. However, the development of circuits that aim to surmount other bottlenecks in cancer immunotherapy remains less advanced. We envisage the next generation of gene circuits will be able to (1) augment T-cell trafficking and accumulation at the tumor site; (2) endow T cells with the ability to discern their location within the body; (3) enable T cells to perceive the suppressive TME and counteract effectively; (4) enhance T-cell proliferation and longevity; (5) prevent T-cell exhaustion; and (6) promote the differentiation of effector cells into memory T cells. These technical advancements will unleash the full potential of CAR-T cells to combat malignancies.

### Opportunities in cancer-targeting gene circuit therapy

Presently, most gene circuit studies have focused on exploring new circuit designs and optimizing circuit performance for superior tumor-targeting specificity. There is a relative dearth of research aimed at harnessing circuit design to enhance therapeutic efficacy and decrease systemic toxicity. We envision that the next generation of gene circuits will possess the ability to (1) target clinically relevant heterogeneous tumor cell populations; (2) eradicate metastasis through systemic delivery or systemic therapeutic responses triggered by local delivery; (3) amplify their therapeutic effects or delivery efficiency locally or systematically; (4) effectively trigger sequential output production; and (5) exhibit minimal off-tumor toxicity throughout the entire body. These advancements are poised to further unlock the complete potential of cancer-targeting gene circuits.
